# Dosimetric verification of annual quality assurance for a linear accelerator using a transmission type detector

**DOI:** 10.1038/s41598-023-45114-2

**Published:** 2023-10-21

**Authors:** Dong Hyeok Choi, Jin Sung Kim, Rena Lee, So Hyun Ahn, Woo Sang Ahn

**Affiliations:** 1 Department of Medicine, Yonsei University College of Medicine, Seoul, South Korea; 2 Medical Physics and Biomedical Engineering Lab (MPBEL), Yonsei University College of Medicine, Seoul, South Korea; 3 Department of Radiation Oncology, Yonsei Cancer Center, Heavy Ion Therapy Research Institute, Yonsei University College of Medicine, Seoul, South Korea; 4 Department of Biomedical Engineering, School of Medicine, Ewha Womans University, Seoul, South Korea; 5 Ewha Medical Research Institute, School of Medicine, Ewha Womans University, Seoul, South Korea; 6 Department of Radiation Oncology, Gangneung Asan Hospital, University of Ulsan College of Medicine, Gangneung, South Korea

**Keywords:** Health care, Health occupations, Medical research

## Abstract

The purpose of our study is to establish an efficient quality assurance (QA) procedure using a transmission-type detector (IBA, Stealth chamber), a reference signal detector, as a field chamber. Relative dosimetry items, including monitor unit linearity, output constancy based on dose rate and field size, and output factor were measured and compared with results obtained from the Farmer-type chamber (IBA, Wellhofer, FC65-G). Moreover, output for each field size was measured to assess its applicability to small fields. Results using the Stealth chamber were in good agreement with the FC65-G within 1.0%, except for output constancy according to gantry angle, which had a 1.1% error rate for the Stealth chamber and 2.7% for the FC65-G. Differences of up to − 6.26% output factor were observed for the Stealth chamber and up to − 0.56% for the CC-13 ionization chamber (IBA) in the 3 × 3 cm^2^ field. Our study confirmed the possibility of using Stealth chambers for relative dosimetry measurement in QA.

## Introduction

To optimize treatment outcomes and patient safety, the performances of linear accelerators for radiotherapy should be dosimetrically and mechanically checked^1–3^. For this purpose, the American Association of Physicists in Medicine recommends performing periodic quality assurance (QA), and their report contains guidelines for recommended QA items and tolerances^4, 5^.

The annual QA has the largest number of items and the strictest tolerances relative to daily or monthly QA in the evaluation of the beam output. Additionally, the American Association of Physicists in Medicine Task Group (TG) 142 suggests that annual QA should be performed on all energy used^5^. For this reason, annual QA is one of the more time-consuming and labor-intensive tasks for medical physicists.

In recent years, the energies of X-rays and electron beams on linear accelerators have become increasingly diversified. Using various energies in treatment planning can create a more precise dose distribution than using a more limited number of energies. In the past, linear accelerators with two or more X-ray energies were common; however, hospitals have recently been using linear accelerators with five–six X-ray energies, including flattening filter-free (FFF) beams, to enable optimal treatment planning^6–9^. An increase in the number of energies of the linear accelerator results in an increase in the time required for performing QA.

The dosimetric QA items can be divided into absolute and relative dosimetry. The absolute dosimetry items are measured using a Farmer-type chamber (Wellhofer FC65-G) and water phantom according to reference or standard dosimetry protocols, such as TG-51 or TRS-398^10, 11^. However it is not necessary to measure relative dosimetry with the standard protocols. For example, the output constancy according to gantry angle can be measured through an in-air setup.

These relative dosimetry evaluate deviation from the reference value. Therefore it is essential to minimize setup such as centering or alignment with machine isocenter^12–14^. This extensive task of increasing the setup accuracy is another contributor to the time-consuming and labor-intensive nature of the QA process.

The standard reference chamber, the Stealth chamber (IBA Dosimetry. GmbH, Schwarzenbruck, Germany) mounted to the gantry head of linear accelerator and do not need to be repositioned, which is particularly useful in small-field dosimetry^15^. Therefore, an endeavor was made to utilize the Stealth chamber both as a reference and field chamber. In the context of this research, a comparative analysis was carried out for various relative dosimetry parameters, comparing the results obtained from the Stealth chamber with those from the FC65-G ionization chamber as part of the annual QA assessment. This evaluation aimed to assess the feasibility of this approach and to analyze any discernible advantages or distinctive characteristics associated with the use of the Stealth chamber in this expanded capacity.

## Materials and methods

### Stealth chamber

The Stealth chamber is used as a reference signal chamber when measuring the percent depth dose or profiles of a linear accelerator^16^.

**Figure 1:**
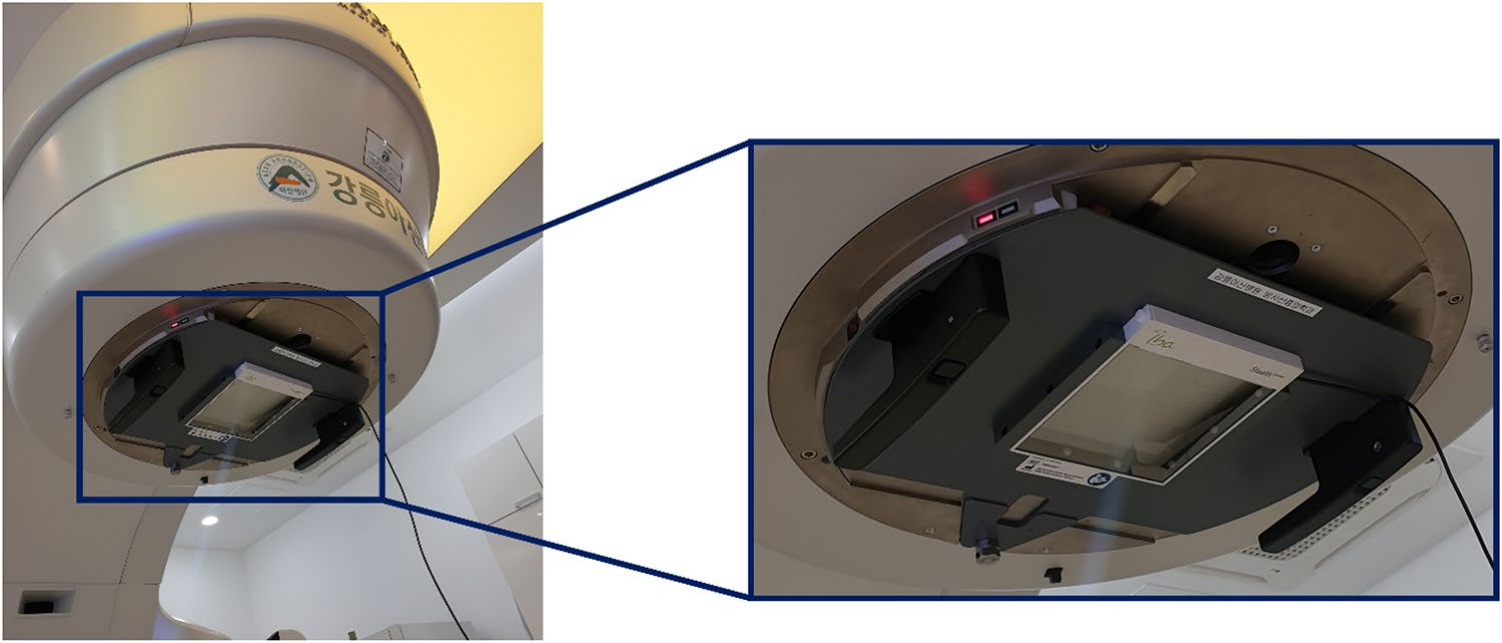
The transmission-type detector (Stealth chamber) attached to the gantry head. Depending on the manufacturer of the linear accelerator, different mounting parts are provided.

Figure 1 shows a Stealth chamber mounted on the gantry of a linear accelerator (Varian TrueBeam, Varian Medical Systems, Palo Alto, CA, USA). In this study, relative dose measurements were performed using the Stealth chamber as a field chamber rather than as a reference signal detector. The Stealth chamber has an active area of 21.9 × 21.9 cm^2^ and can be used for field sizes ranging from 0.5 × 0.5 cm^2^ to 20.0 × 20.0 cm^2^. The Stealth chamber's optical transparency is over 75%, and it is made of Lexan 9030 material (density = 1.2 g/cm^3^), about 3 mm thick, and weighs about 2150 g.

### Measured QA items

The measurements were performed for the annual QA items recommended in the report of the AAPM TG 142 as shown in Table 1. The items were relative dosimetry measurements that could be performed using the Stealth chamber.

**Table 1:** Measured annual QA items recommended by the AAPM TG 142 and tolerance criteria for non-intensity modulated radiation therapy (IMRT), IMRT, and stereotactic radiosurgery (SRS)/stereotactic body radiotherapy (SBRT).

Procedure	Machine-type tolerance		
	Non-IMRT	IMRT	SRS/SBRT
X-ray monitor unit linearity (output constancy)	± 2% ≥ 5 MU	± 5% (2–4 MU), ± 2% ≥ 5 MU	± 5% (2–4 MU) ± 2% ≥ 5 MU
Electron monitor unit linearity (output constancy)		± 2% (2–4 MU) ≥ 5 MU	
X-ray output constancy versus dose rate		± 2% from baseline	
X-ray output constancy versus gantry angle		± 1% from baseline	
Electron output constancy vs gantry angle		± 1% from baseline	
Output factor for X-ray	2% for field size $<$ 4 × 4 cm^2^, 1% $\ge$ 4 × 4 cm^2^		

### Experimental conditions

The evaluation of output involved a thorough analysis utilizing the energy and MU predominantly employed at our institution. Additionally, various dose rates were used to test the clinically used dose rates. In the IMRT beam, less than 4 MU is rarely used, so more than 4 MU was used. In the case of the electron beam, it was not measured at 180° due to the influence of the couch collision.

The field size was set to 10 × 10 cm^2^, and means and standard deviations were calculated for triplicate measurements. Deviations were calculated based on the values measured at each baseline.

Prior to utilizing the Stealth chamber for dose measurement, an evaluation of dose rate dependence was conducted to ascertain minimal influence from dose rate-dependent effects. It was intended to shorten the measurement time by irradiating the beam by setting the MU transfer to a time that falls to an integer. Accordingly, measurements were performed by selecting different optimal dose rates and times for each detector.

Measurements for both the Stealth chamber and the FC65-G were carried out simultaneously to mitigate daily variations, particularly those pertaining to dose rate. Furthermore, the reproducibility of the dose rate was verified to fall within a 1% margin of error. Efforts were made to align the temperature and pressure conditions as closely as feasible to reduce potential errors in the measurement outcomes.

#### X-ray and electron MU linearity

The setup conditions for the Stealth chamber and FC65-G were the same: seven X-ray energies of 4 MV, 6 MV, 8 MV, 10 MV, 15 MV, 6 MV FFF, and 10 MV FFF were measured. The MUs were set to 4, 7, 15, 50, 100, and 300 MU, and the dose rates for 4 MV, 6 MV FFF, and 10 MV FFF were set to 250, 800, and 800 MU/min. For the remaining energies, a dose rate of 400 MU/min was used.

In the case of electron beams, measurements were performed for the energies of 6 MeV, 9 MeV, 12 MeV, 15 MeV, 16 MeV, and 20 MeV. The MUs were set to 4, 7, 15, 50, 100, and 300 MU for the Stealth chamber and 7, 15, 50, 100, and 300 MU for the FC65-G. For all energies of the electron beam, dose rates of 400 MU/min for the Stealth chamber and 500 MU/min for the FC65-G were used.

The field size was set to 10 × 10 cm^2^, and means and standard deviations were calculated for triplicate measurements. The differences from baseline were evaluated to verify whether they were within the tolerance range. The R-squared (R^2^) value was used to quantitatively evaluate MU linearity.

#### X-ray output constancy with dose rate

For Stealth chamber and FC65-G measurements, the output variation was measured by changing the dose rate from 100 to 600 MU/min for flattening-filter X-ray energies and from 400 to 2400 MU/min for FFF X-ray energies. For the Stealth chamber, 100 MU for flattening-filter X-ray energies and 500 MU for FFF X-ray energies were delivered. For the FC65-G, 80 MU was delivered. The field size was set to 10 × 10 cm^2^, and means and standard deviations were calculated for triplicate measurements. The differences from baseline were evaluated to verify whether they were within tolerance ranges.

#### X-ray and electron output constancy with gantry angle

Output deviation was measured at the following gantry angles: 0°, 90°, 180°, and 270° for X-ray and 0°, 90°, and 270° for the electron energies. The field sizes were set to 25 × 25 cm^2^ for X-ray and 10 × 10 cm^2^ for the electron beams. Dose rates 400 and 500 MU/min were used for the Stealth chamber and FC65-G measurements. Means and standard deviations were calculated for triplicate measurements. The differences from baseline were evaluated to verify whether they were within tolerance ranges.

#### X-ray constancy with field size

The measured charge is dependent of the applied field size. Output measurement by field size was performed using Stealth chamber and CC-13 ionization chamber in TrueBeam. Stealth chamber measurements were conducted by installing the detector in the gantry head, while CC-13 ionization chamber measurements were performed using a solid water phantom. The dose rate, MU, and source-to-axis distance (SAD), Whether to use flattening filter set up for each equipment are shown in Table 2. In addition, the output factor acquired by Radiation Treatment Planning System (RTPS) and the output factor for each field size calculated by the Stealth chamber were compared. The output factor for the field size in the measuring institution was obtained using CC-13 ionization chamber at the time of installation of the TrueBeam.

**Table 2:** Information on use of dose rate, MU, SAD, flattening filter for output measurement of field size.

Test conditions	Chamber	
	Stealth chamber	CC-13 ionization chamber
Dose rate (MU/min)	400	400
MU	100	100
SAD (cm)	Installed directly on gantry head	100 (Depth 5 cm)
Flattening filter	FF/FFF	FF/FFF

*FFF Flattening filter free.

### Statistical analysis

A two-sample t-test for independent samples was conducted to assess the presence of a significant disparity in the output measurements the Stealth chamber and FC65-G each QA item. The null hypothesis (H0) posited that no substantial difference exists in the measurements between the two chambers, while the alternative hypothesis (H1) proposed the presence of a significant difference. The test was performed at a significance level (α) of 0.05, indicating a 5% probability of committing a Type I error.

Prior to the t-test, several prerequisites were verified. First, each measurement within one chamber was independent of those within the other chamber was assumed. Subsequently, the variances of the population distributions were compared using the F test.

Following the t-test analysis, the null hypothesis (H0) was either retained or refuted based on the calculated results. Specifically, when the *p*-value was less than 0.05, it was indicative of a noteworthy distinction in the output measurements between Stealth chamber and FC65-G. In such cases, the null hypothesis was rejected in favor of the alternative hypothesis.

## Results

### X-ray and electron MU linearity

**Figure 2:**
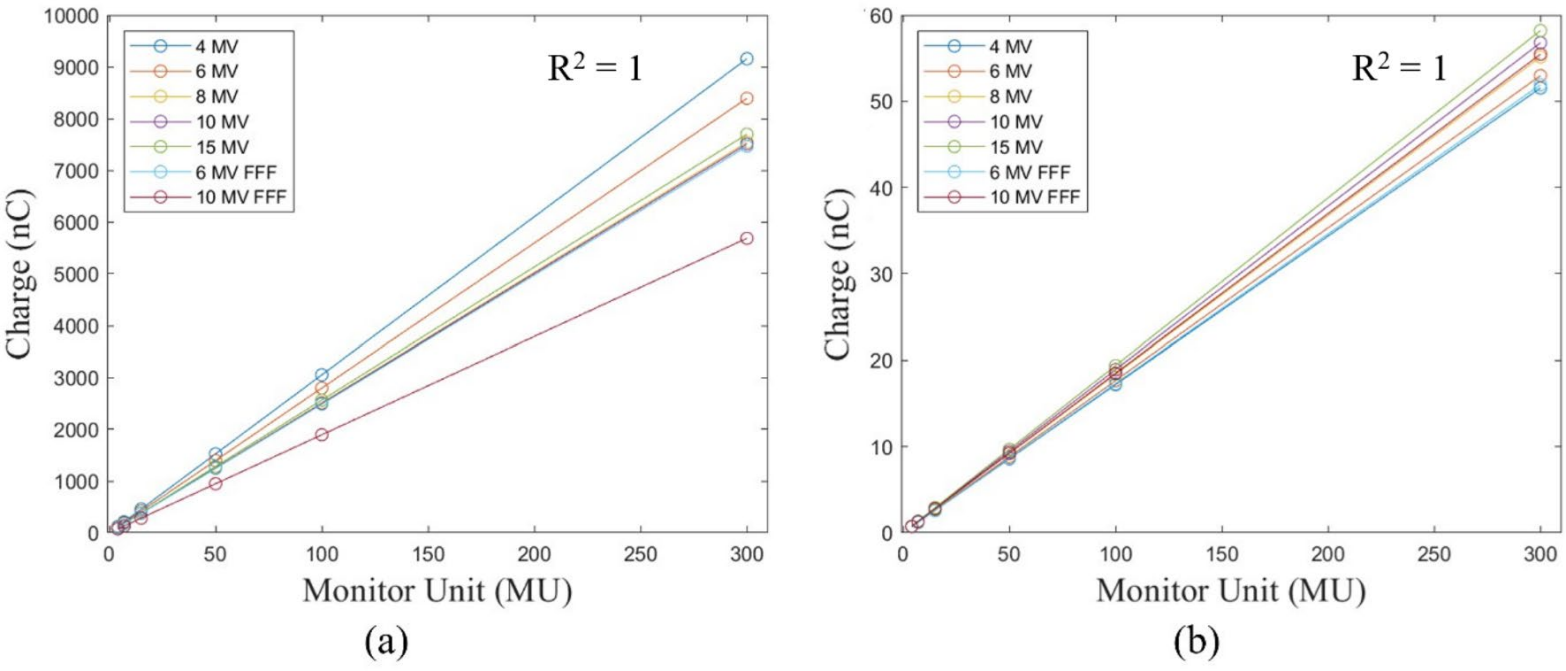
Relationship between X-ray MU and collected charge with (**a**) Stealth chamber, and (**b**) FC65-G.

Table 3 shows the X-ray MU linearity exhibited deviations from the baseline value from − 0.82 to 0.07% and from − 0.17 to 0.96% for the Stealth chamber and FC65-G. In both chamber types, Fig. 2 displays the R^2^ value for the linearity slope in relation to the change in MU.

**Table 3:** X-ray output linearity of the different MUs with reference to the measured at 100 MU.

Energies	FF/FFF	MUs	Stealth chamber		FC65-G	
			Mean ± STD (nC)	Baseline	Mean ± STD (nC))	Baseline (%)
4 MV	FF	4	122.05 ± 0.01	− 0.07%	0.69 ± 0.00	− 0.17
		7	213.61 ± 0.01	− 0.06%	1.20 ± 0.00	0.09
		15	457.70 ± 0.04	− 0.06%	2.58 ± 0.00	− 0.05
		50	1525.66 ± 0.04	− 0.06%	8.59 ± 0.00	− 0.02
		100	3053.28 ± 0.22	0.00%	17.19 ± 0.00	0.00
		300	9155.05 ± 1.43	− 0.05%	51.54 ± 0.00	− 0.03
6 MV	FF	4	111.81 ± 0.02	− 0.09%	0.71 ± 0.00	− 0.06
		7	195.60 ± 0.04	− 0.13%	1.24 ± 0.00	− 0.04
		15	419.29 ± 0.04	− 0.09%	2.65 ± 0.00	− 0.08
		50	1397.21 ± 0.07	− 0.12%	8.82 ± 0.00	− 0.02
		100	2797.80 ± 0.30	0.00%	17.65 ± 0.00	0.00
		300	8390.94 ± 2.09	− 0.03%	52.98 ± 0.02	0.05
8 MV	FF	4	100.54 ± 0.02	− 0.02%	0.74 ± 0.00	0.07
		7	175.94 ± 0.03	− 0.03%	1.29 ± 0.00	0.06
		15	377.09 ± 0.00	− 0.01%	2.76 ± 0.00	− 0.05
		50	1256.51 ± 0.07	− 0.04%	9.19 ± 0.00	− 0.01
		100	2514.08 ± 0.09	0.00%	18.38 ± 0.00	0.00
		300	7542.92 ± 0.56	0.01%	55.14 ± 0.00	0.05
10 MV	FF	4	100.06 ± 0.01	− 0.04%	0.76 ± 0.00	0.25
		7	175.13 ± 0.02	− 0.02%	1.33 ± 0.00	0.08
		15	375.26 ± 0.03	− 0.02%	2.84 ± 0.00	0.07
		50	1250.81 ± 0.14	− 0.03%	9.46 ± 0.00	0.01
		100	2502.30 ± 0.13	0.00%	18.92 ± 0.00	0.00
		300	7507.64 ± 0.51	0.01%	56.79 ± 0.01	0.05
15 MV	FF	4	102.63 ± 0.05	0.03%	0.78 ± 0.00	0.87
		7	179.62 ± 0.00	0.04%	1.36 ± 0.00	0.42
		15	381.59 ± 5.76	− 0.82%	2.92 ± 0.00	0.22
		50	1282.85 ± 0.13	− 0.03%	9.70 ± 0.00	0.06
		100	2564.95 ± 0.19	0.00%	19.39 ± 0.00	0.00
		300	7697.42 ± 0.71	0.03%	58.2 ± 0.00	0.05
6 MV	FFF	4	99.38 ± 0.36	− 0.10%	0.70 ± 0.00	0.74
		7	174.01 ± 0.07	− 0.05%	1.21 ± 0.00	0.25
		15	373.33 ± 0.02	0.07%	2.60 ± 0.00	0.02
		50	1243.64 ± 0.24	0.01%	8.65 ± 0.00	0.05
		100	2487.10 ± 0.11	0.00%	17.30 ± 0.00	0.00
		300	7461.58 ± 0.71	0.03%	51.92 ± 0.01	0.03
10 MV	FFF	4	75.62 ± 0.16	− 0.24%	0.74 ± 0.00	0.43
		7	131.92 ± 0.28	− 0.56%	1.31 ± 0.00	0.96
		15	284.37 ± 0.50	− 0.03%	2.79 ± 0.00	0.41
		50	947.99 ± 0.18	− 0.04%	9.23 ± 0.00	− 0.15
		100	1895.21 ± 0.65	0.00%	18.49 ± 0.00	0.00
		300	5685.26 ± 0.64	− 0.0001	55.46 ± 0.00	− 0.02

*STD: standard deviation.

**Figure 3:**
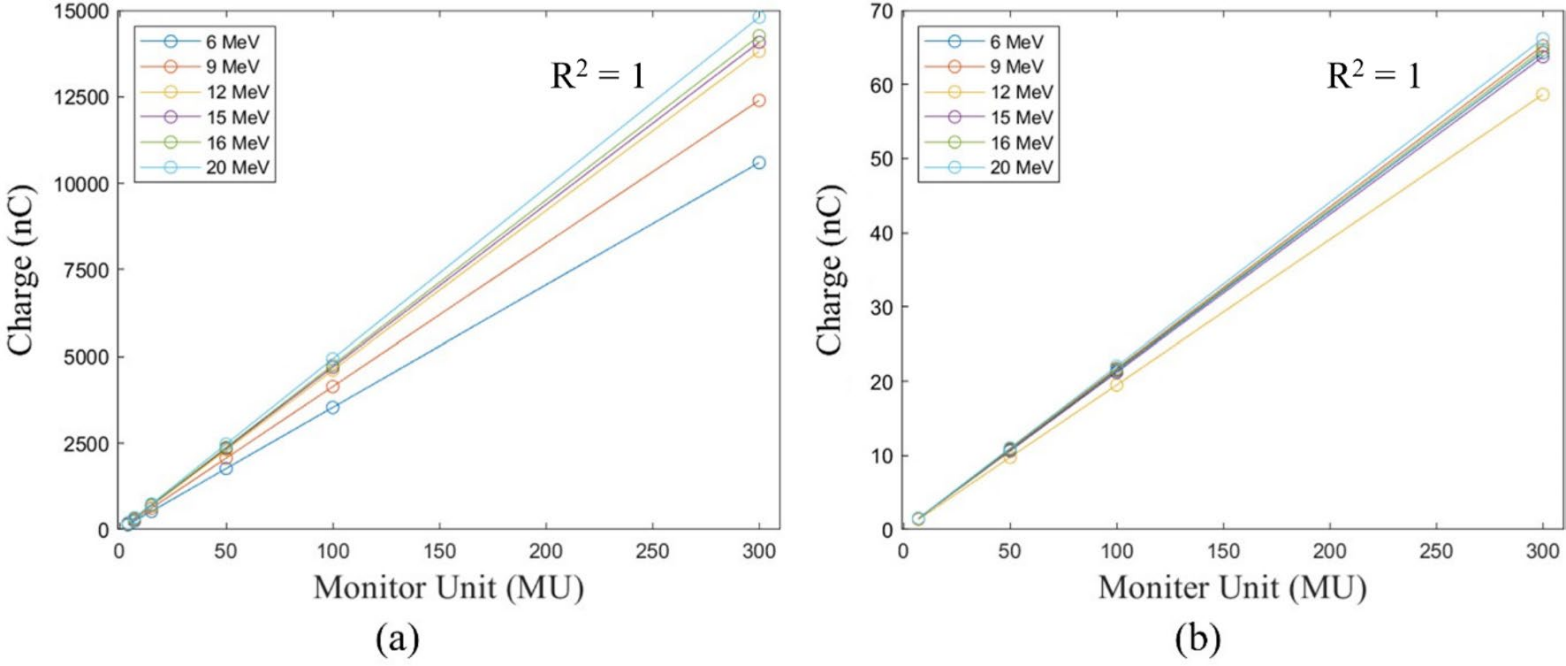
Relationship between the electron MU and collected charge with (**a**) Stealth chamber, and (**b**) FC65-G.

Table 4 shows the electron MU linearity exhibited deviations from the baseline value from − 0.63 to 0.43% and from − 0.01 to 0.70% for the Stealth chamber and FC65-G. In both chamber types, Fig. 3 displays the R^2^ value for the linearity slope in relation to the change in MU.

**Table 4:** Electron output linearity of the different MUs with reference to the measured at 100 MU.

Energy	MU	Stealth chamber		FC65-G	
		Mean	Baseline (%)	Mean	Baseline (%)
6 MeV	4	140.40 ± 0.22	− 0.64		
	7	247.43 ± 0.82	0.06	1.50 ± 0.00	− 0.01
	15	529.66 ± 0.79	− 0.04		
	50	1766.92 ± 0.23	0.04	10.73 ± 0.00	0.09
	100	3532.51 ± 0.62	0.00	21.43 ± 0.00	0.00
	300	10,596.25 ± 0.77	− 0.01	64.29 ± 0.01	0.00
9 MeV	4	165.70 ± 0.59	0.21		
	7	289.63 ± 0.07	0.08	1.52 ± 0.00	0.14
	15	619.92 ± 0.83	− 0.03		
	50	2066.69 ± 0.93	− 0.02	10.88 ± 0.01	0.14
	100	4134.13 ± 1.48	0.00	21.72 ± 0.01	0.00
	300	12,396.41 ± 0.78	− 0.05	65.20 ± 0.00	0.06
12 MeV	4	185.06 ± 0.97	0.43		
	7	322.30 ± 0.26	− 0.05	1.37 ± 0.00	0.10
	15	690.99 ± 1.03	0.00		
	50	2302.43 ± 0.10	− 0.03	9.77 ± 0.00	0.16
	100	4606.36 ± 1.16	0.00	19.51 ± 0.00	0.00
	300	13,814.63 ± 2.64	− 0.03	58.65 ± 0.02	0.21
15 MeV	4	188.30 ± 1.19	0.27		
	7	329.50 ± 1.01	0.26	1.49 ± 0.00	0.21
	15	704.27 ± 0.21	0.01		
	50	2348.09 ± 0.99	0.03	10.62 ± 0.00	0.17
	100	4694.88 ± 1.08	0.00	21.21 ± 0.00	0.00
	300	14,076.71 ± 0.96	− 0.06	63.74 ± 0.01	0.19
16 MeV	4	190.41 ± 1.61	0.04		
	7	333.41 ± 1.79	0.10	1.52 ± 0.00	0.70
	15	713.30 ± 1.30	− 0.06		
	50	2377.97 ± 0.44	− 0.05	10.79 ± 0.00	0.09
	100	4758.14 ± 0.19	0.00	21.55 ± 0.01	0.00
	300	14,265.43 ± 2.87	− 0.06	64.69 ± 0.00	0.05
20 MeV	4	196.94 ± 0.62	− 0.21		
	7	345.31 ± 0.36	− 0.02	1.55 ± 0.00	0.57
	15	740.15 ± 0.63	0.00		
	50	2470.05 ± 1.58	0.12	11.02 ± 0.00	0.07
	100	4934.15 ± 0.91	0.00	22.03 ± 0.01	0.00

### X-ray output constancy with dose rate

**Figure 4:**
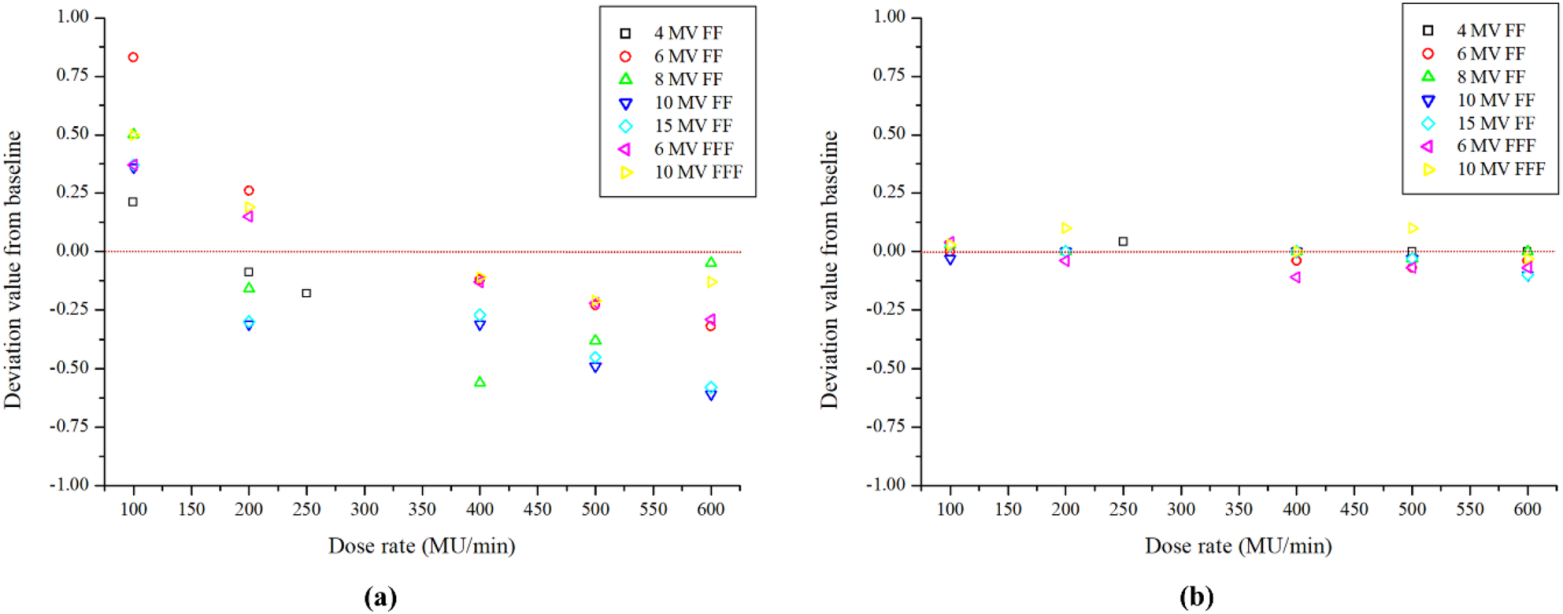
Deviation in the detector when measuring X-ray output consistency of the different dose rate with reference to the measured at 300 MU and 150 MU. (**a**) Stealth chamber, and (**b**) FC65-G.

Table 5 presents the constancy of X-ray output at various dose rates, referencing measurements taken at 300 MU and 150 MU. Figure 4 displays deviations from the reference measurements, ranging from − 0.61 to 0.83% for the Stealth chamber and − 0.11 to 0.10% for the FC65-G.

**Table 5:** X-ray output constancy of the different dose rate with reference to the measured at 300 MU, and 150 MU.

Energy	FF/FFF	Dose rate	Stealth chamber		FC65-G chamber	
			Mean ± STD (nC)	Baseline (%)	Mean ± STD (nC)	Baseline (%)
4 MV	FF	100	2349.47 ± 4.72	0.21	13.75 ± 0.00	0.00
		150	2323.12 ± 0.45	0.00	13.75 ± 0.00	0.00
		200	2311.63 ± 0.35	− 0.09	13.75 ± 0.00	0.00
		250	2300.63 ± 0.61	− 0.18	13.76 ± 0.01	0.04
6 MV	FF	100	2849.65 ± 0.45	0.83	14.12 ± 0.00	0.00
		200	2833.40 ± 0.29	0.26	14.12 ± 0.00	0.00
		300	2826.17 ± 1.21	0.00	14.12 ± 0.00	0.00
		400	2822.69 ± 0.04	− 0.12	14.12 ± 0.01	− 0.04
		500	2819.68 ± 0.22	− 0.23	14.11 ± 0.00	− 0.07
		600	2817.13 ± 0.32	− 0.32	14.12 ± 0.01	− 0.04
8 MV	FF	100	2577.35 ± 0.45	0.50	14.71 ± 0.01	0.03
		200	2560.36 ± 0.29	− 0.16	14.70 ± 0.00	0.00
		300	2564.44 ± 1.21	0.00	14.70 ± 0.00	0.00
		400	2550.02 ± 0.04	− 0.56	14.70 ± 0.00	0.00
		500	2554.76 ± 0.22	− 0.38	14.70 ± 0.01	− 0.03
		600	2551.62 ± 0.32	− 0.05	14.70 ± 0.00	0.00
10 MV	FF	100	2562.72 ± 0.19	0.36	15.15 ± 0.01	− 0.03
		200	2545.71 ± 0.29	− 0.31	15.15 ± 0.01	0.00
		300	2553.58 ± 0.32	0.00	15.15 ± 0.00	0.00
		400	2545.69 ± 0.22	− 0.31	15.14 ± 0.00	0.00
		500	2541.12 ± 0.07	− 0.49	15.14 ± 0.01	− 0.03
		600	2538.04 ± 0.26	− 0.61	15.14 ± 0.00	− 0.10
15 MV	FF	100	2621.94 ± 0.24	0.37	15.52 ± 0.01	0.03
		200	2604.61 ± 0.20	− 0.30	15.51 ± 0.00	0.00
		300	2612.35 ± 0.32	0.00	15.51 ± 0.00	0.00
		400	2605.36 ± 0.22	− 0.27	15.51 ± 0.00	0.00
		500	2600.72 ± 0.07	− 0.45	15.51 ± 0.01	− 0.03
		600	2597.25 ± 0.26	− 0.58	15.50 ± 0.01	− 0.10
6 MV	FFF	100	2621.94 ± 3.42	0.37	13.85 ± 0.00	0.04
		200	2604.61 ± 2.32	0.15	13.84 ± 0.00	− 0.04
		300	2612.35 ± 2.19	0.00	13.85 ± 0.01	0.00
		400	2605.36 ± 0.61	− 0.13	13.83 ± 0.00	− 0.11
		500	2600.72 ± 1.47	− 0.22	13.84 ± 0.01	− 0.07
		600	2597.25 ± 2.32	− 0.29	13.84 ± 0.01	− 0.07
10 MV	FFF	100	9567.16 ± 0.62	0.50	14.79 ± 0.01	0.03
		200	9536.33 ± 0.63	0.19	14.80 ± 0.01	0.10
		300	9518.70 ± 0.69	0.00	14.79 ± 0.01	0.00
		400	9508.41 ± 0.08	− 0.11	14.79 ± 0.01	0.00
		500	9498.64 ± 0.95	− 0.21	14.80 ± 0.00	0.10
		600	9505.94 ± 2.53	− 0.13	14.78 ± 0.00	− 0.03

The difference from baseline was evaluated using the reference dose rate as the baseline.

### X-ray and electron output constancy with gantry angle

**Figure 5:**
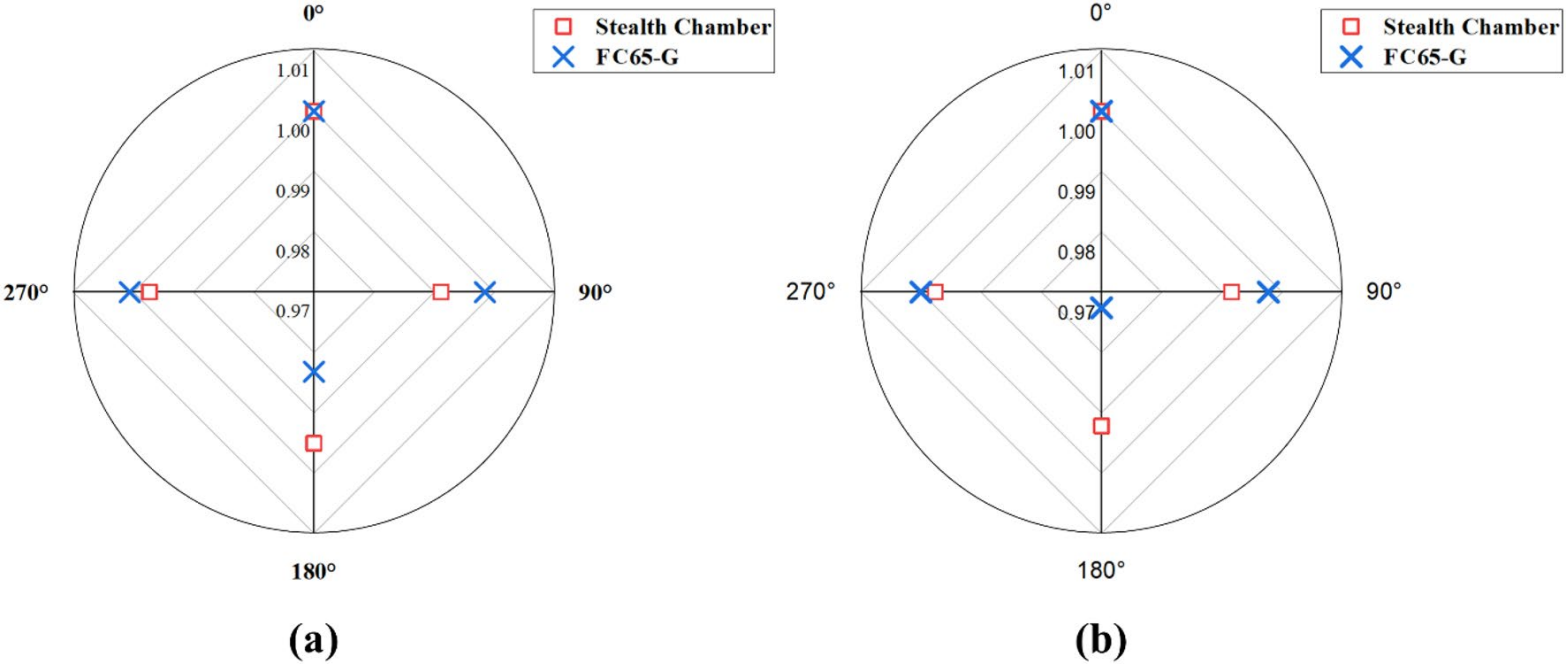
Deviation in the detector when measuring X-ray output constancy with gantry angle for baseline: (**a**) The smallest deviation at 15 MV FF, and (**b**) The largest deviation at 4 MV FF.

Table 6 shows the X-ray output constancy in relation to gantry angle deviations, referencing readings measured at a gantry angle of 0°. Additionally, Fig. 5 presents the minimum and maximum deviations, which were − 1.07 to 0.00% for the Stealth chamber and − 2.74 to 0.28% for the FC65-G.

**Table 6:** X-ray output constancy of the different gantry angles with reference to readings measured at gantry angle of 0°.

Energy	FF/FFF	Gantry angle (°)	Stealth chamber		FC65-G	
			Mean ± STD (nC)	Baseline (%)	Mean ± STD (nC)	Baseline (%)
4 MV	FF	0	1530.24 ± 0.04	0.00	13.13 ± 0.00	0.00
		90	1517.38 ± 0.10	− 0.84	13.10 ± 0.00	− 0.23
		180	1518.36 ± 0.05	− 0.78	12.77 ± 0.00	− 2.74
		270	1526.49 ± 0.43	− 0.24	13.13 ± 0.00	0.00
6 MV	FF	0	1405.48 ± 0.10	0.00	13.83 ± 0.00	0.00
		90	1392.92 ± 0.15	− 0.89	13.80 ± 0.00	− 0.25
		180	1393.43 ± 0.16	− 0.86	13.50 ± 0.00	− 2.42
		270	1400.20 ± 0.06	− 0.38	13.83 ± 0.00	0.00
8 MV	FF	0	1270.80 ± 0.06	0.00	14.57 ± 0.00	0.00
		90	1257.14 ± 0.03	− 1.07	14.55 ± 0.00	− 0.17
		180	1260.27 ± 0.05	− 0.83	14.30 ± 0.00	− 1.89
		270	1264.65 ± 0.06	− 0.48	14.60 ± 0.00	0.17
10 MV	FF	0	1266.66 ± 0.25	0.00	15.16 ± 0.00	0.00
		90	1254.88 ± 0.08	− 0.93	15.13 ± 0.00	− 0.20
		180	1258.58 ± 0.09	− 0.64	14.90 ± 0.00	− 1.75
		270	1262.36 ± 0.13	− 0.34	15.17 ± 0.00	0.07
15 MV	FF	0	1296.84 ± 0.24	0.00	15.77 ± 0.00	0.00
		90	1285.26 ± 0.11	− 0.89	15.75 ± 0.00	− 0.16
		180	1290.53 ± 0.08	− 0.49	15.51 ± 0.00	− 1.68
		270	1293.38 ± 0.03	− 0.27	15.78 ± 0.00	0.06
6 MV	FFF	0	1259.02 ± 0.26	0.00	13.17 ± 0.00	0.00
		90	1248.26 ± 0.22	− 0.85	13.16 ± 0.00	− 0.11
		180	1248.07 ± 0.11	− 0.87	12.84 ± 0.00	− 2.54
		270	1254.52 ± 0.14	− 0.36	13.20 ± 0.00	0.19
10 MV	FFF	0	957.69 ± 0.28	0.00	14.54 ± 0.00	0.00
		90	948.40 ± 0.19	− 0.97	14.54 ± 0.00	0.00
		180	949.93 ± 0.43	− 0.81	14.27 ± 0.00	− 1.86
		270	953.30 ± 0.26	− 0.46	14.58 ± 0.00	0.28

The difference from baseline was evaluated using the reference dose rate as the baseline.

**Figure 6:**
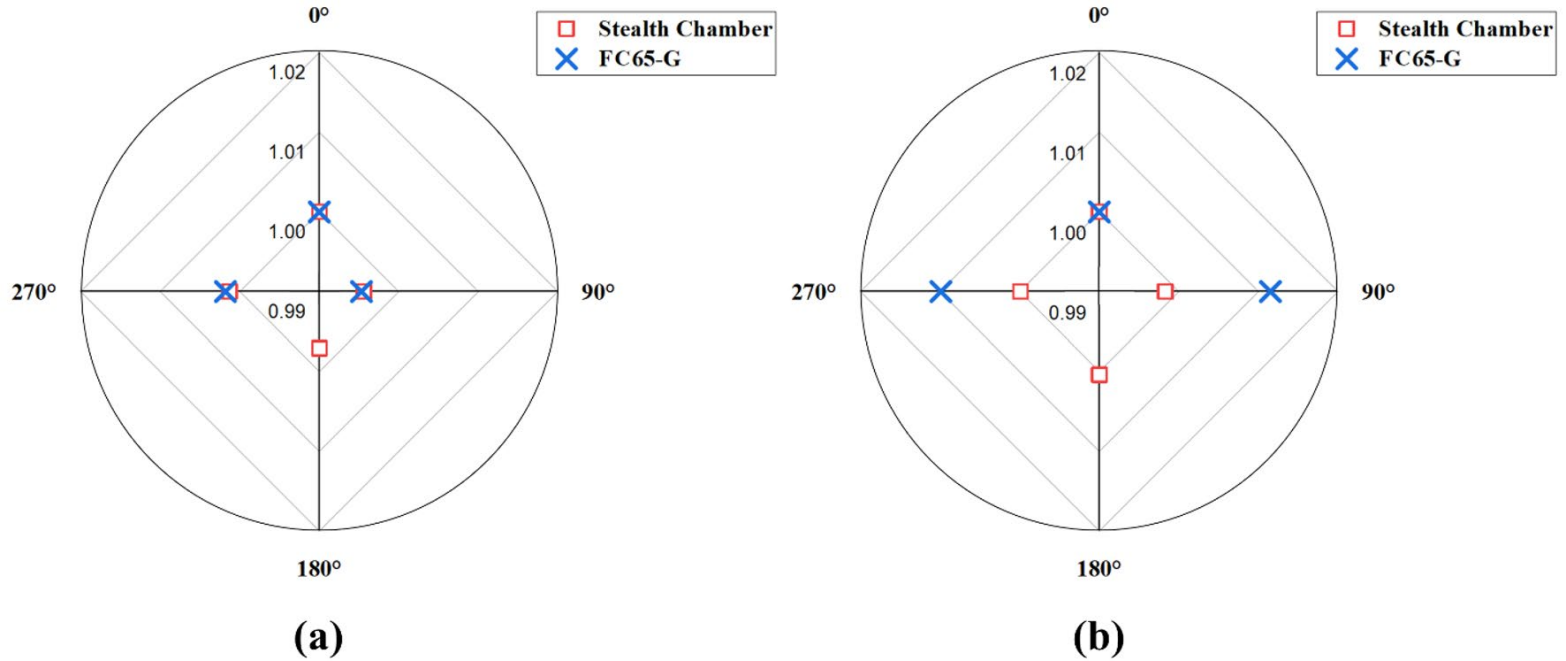
Deviation in the detector when measuring electron output constancy with gantry angle for the baseline: (**a**) the smallest deviation at 20 MeV, and (**b**) the largest deviation at 15 MeV.

Table 7 shows the electron output constancy in relation to gantry angle deviations, referencing readings measured at a gantry angle of 0°. Furthermore, Fig. 6 illustrates the minimum and maximum deviations, ranging from − 0.44 to 0.44% for the Stealth chamber and − 0.69 to 1.36% for the FC65-G.

**Table 7:** Electron output constancy of the different gantry angles with reference to readings measured at gantry angle of 0°.

Energy	Gantry angle (°)	Stealth chamber		FC65-G	
		Mean ± STD (nC)	Baseline (%)	Mean ± STD (nC)	Baseline (%)
6 MeV	0	1789.97 ± 0.31	0.00	0.33 ± 0.00	0.00
	90	1783.43 ± 0.47	− 0.37	0.33 ± 0.00	− 0.69
	180	1788.16 ± 0.85	− 0.10		
	270	1797.86 ± 0.62	0.44	0.33 ± 0.00	0.70
9 MeV	0	2094.78 ± 0.67	0.00	0.55 ± 0.00	0.00
	90	2088.91 ± 0.31	− 0.28	0.55 ± 0.00	0.28
	180	2093.45 ± 0.25	− 0.06		
	270	2100.33 ± 0.67	0.26	0.56 ± 0.00	0.94
12 MeV	0	2330.43 ± 0.43	0.00	1.07 ± 0.00	0.00
	90	2321.96 ± 0.78	− 0.36	1.08 ± 0.00	0.93
	180	2325.30 ± 0.53	− 0.22		
	270	2335.93 ± 0.64	0.24	1.08 ± 0.00	1.36
15 MeV	0	2368.96 ± 1.16	0.00	4.37 ± 0.00	0.00
	90	2364.81 ± 0.37	− 0.17	4.42 ± 0.01	1.15
	180	2369.98 ± 1.41	0.04		
	270	2368.62 ± 1.84	− 0.01	4.42 ± 0.00	0.99
16 MeV	0	2405.01 ± 0.68	0.00	9.35 ± 0.00	0.00
	90	2397.03 ± 0.12	− 0.33	9.4 ± 0.02	0.51
	180	2401.94 ± 1.13	− 0.13		
	270	2411.71 ± 0.79	0.28	9.38 ± 0.00	0.29
20 MeV	0	2493.94 ± 1.66	0.00	13.94 ± 0.00	0.00
	90	2483.08 ± 1.10	− 0.44	13.88 ± 0.01	− 0.47
	180	2486.62 ± 1.70	− 0.29		
	270	2497.26 ± 1.12	0.13	13.97 ± 0.01	0.18

The difference from baseline was evaluated using the reference dose rate as the baseline.

### Output factor for X-ray

Deviations were obtained for the X-ray output, considering a field size of 10 × 10 cm^2^. Both chambers exhibited a trend of decreasing output factors with a reduction in field size.

The output factor of the Stealth chamber used to RTPS, there was a difference of up to − 6.26% in the FFF beam, and up to − 5.45% in the FF beam.

In the case of the output factor of the CC-13 ionization chamber used to initially install the equipment, there was a difference of up to − 0.56% in the FFF beam, and up to − 0.53 in the FF beam (Table 8).

**Table 8:** The X-ray output constancy with the field size in TrueBeam.

FF/FFF	Energy	Field size	Stealth chamber			CC-13 ionization chamber		
			OF	OF (RTPS)	Diff. (%)	OF	OF (RTPS)	Diff. (%)
FFF	6	3 × 3	0.84	0.90	− 6.26	0.90	0.90	− 0.56
		5 × 5	0.90	0.94	− 3.74	0.94	0.94	0.00
		10 × 10	1.00	1.00	0.00	1.00	1.00	0.00
		20 × 20	1.08	1.05	3.25	1.05	1.05	0.00
		25 × 25	1.11	1.06	4.37	1.06	1.06	0.28
	10	3 × 3	0.89	0.92	− 3.29	0.92	0.92	0.11
		5 × 5	0.94	0.96	− 2.11	0.96	0.96	0.31
		10 × 10	1.00	1.00	0.00	1.00	1.00	0.00
		20 × 20	1.05	1.03	1.72	1.03	1.03	0.00
		25 × 25	1.06	1.04	1.93	1.04	1.04	− 0.19
FF	6	3 × 3	0.83	0.88	− 5.45	0.88	0.88	− 0.23
		5 × 5	0.89	0.93	− 3.90	0.93	0.93	− 0.43
		10 × 10	1.00	1.00	0.00	1.00	1.00	0.00
		20 × 20	1.10	1.07	3.21	1.07	1.07	− 0.09
		25 × 25	1.14	1.09	4.31	1.09	1.09	− 0.28
	10	3 × 3	0.85	0.88	− 2.84	0.88	0.88	0.23
		5 × 5	0.91	0.94	− 2.75	0.94	0.94	− 0.53
		10 × 10	1.00	1.00	0.00	1.00	1.00	0.00
		20 × 20	1.08	1.06	1.80	1.06	1.06	− 0.38
		25 × 25	1.10	1.07	3.16	1.07	1.07	0.28
	15	3 × 3	0.86	0.88	− 2.57	0.88	0.88	− 0.45
		5 × 5	0.92	0.94	− 1.99	0.94	0.94	− 0.21
		10 × 10	1.00	1.00	0.00	1.00	1.00	0.00
		20 × 20	1.07	1.05	1.66	1.05	1.05	0.00
		25 × 25	1.09	1.06	2.64	1.06	1.06	0.38

*OF: Output factor.

### Statistical analysis of chamber comparison

The output data obtained from the Stealth chamber and the FC65-G were subjected to an F-test to determine whether they exhibit homoscedasticity or heteroscedasticity. The results confirmed that the two datasets display heteroscedasticity. Subsequently, a heteroscedasticity t-test was conducted, taking into account the observed discrepancies in variance between the chambers. Table 9 provides a summary of the t-test results for output measurements within each chamber corresponding to various QA items.

**Table 9:** T-test results for output values measured in the Stealth chamber and FC65-G as part of the annual QA procedure.

Procedure	*p*-value: result of t-test	Significance between chambers
X-ray monitor unit linearity (output constancy)	0.15	No significant difference
Electron monitor unit linearity (output constancy)	0.18	No significant difference
X-ray output constancy vs dose rate	0.01	Significant difference
X-ray output constancy vs gantry angle	1.97E−08	Significant difference
Electron output constancy vs gantry angle	5.43E−06	Significant difference

## Discussion

The transmission detector used in this study was initially developed as a reference signal chamber for relative dosimetry^17–21^. This detector is used to address the issue of the reference chamber location. In a relative dosimetry system, particularly in small field dosimetry, the reference chamber can cover the field chamber in the same beam direction, which has more serious effects. Another advantage of the transmission detector is that whenever the field size changes during measurement, the operator is not required to enter the treatment room to adjust the position of the reference chamber. This helps to reduce measurement uncertainty due to laser alignment and orientation dependence during QA. Ion chambers may have different isocenter distances. Since the distance difference affects the square of the dose, even a small error has a large effect. However, the stealth is small because the field size is large and the set-up error for the isocenter is attached to the gantry head.

The process of setting up the water phantom for annual QA and the intricate configuration required for measuring beam output typically demands a minimum of one hour. However, in cases where a patient's treatment plan undergoes modifications and there is an urgent need to commence the treatment, the utilization of the Stealth chamber significantly reduces the setup duration, allowing for immediate responses.

There is a research case wherein X-ray output evaluation was performed using a Stealth chamber as a field chamber^22^. In existing study, the Stealth chamber was compared with a FC65-G used as a conventional field chamber. However, only the evaluation of output constancy according to the gantry angle was performed. However, in this study, X-ray and electron MU linearity, X-ray output constancy with dose rate, and X-ray constancy with field size were additionally evaluated.

In another study, a rapid and dependable daily consistency assessment of Volumetric Modulated Arc Therapy (VMAT) was conducted utilizing a Stealth chamber to identify irregularities in beam delivery parameters^23^. The monitoring of VMAT beam consistency, employing the Stealth chamber, corroborated a consistent pattern of change akin to that observed with the Farmer chamber, thus substantiating the potential utility of the Stealth chamber for this purpose. Our study distinguishes itself from the aforementioned research by focusing on assessing the repeatability and constancy of VMAT beams (both clockwise and counterclockwise) with sliding slits, encompassing variations in dose rate, gantry rotation speed, and leaf speed.

De Chavez et al. demonstrated that transmitted chambers, exemplified by the Stealth chamber, constitute a viable alternative to large-area parallel plate chambers for the measurement of dose-area products and the derivation of small field output factors^24^. In our study, the output factors of the Stealth chamber and the CC-13 ionization chamber were acquired and compared, altering the field size. As shown in Table 8, notable stability in response to changes in output factors within small field sizes was exhibited by the Stealth chamber when compared with chambers traditionally used in similar small field configurations.

The Stealth chamber is a gantry-mounted transmission detector with the limitation of attenuation during beam scanning measurements. To measure the percent depth dose using a stealth detector as a reference signal chamber, the effect of attenuation should be considered in the analysis. According to the manufacturer, attenuation by transmission has an effect of approximately 2%^25^. However, in this study, there was no need to consider attenuation because relative dosimetry was performed to observe the deviation between the measured values. Another limitation of the stealth detector is that it can only be used for field sizes ranging from 1 × 1 cm^2^ to 25 × 25 cm^2^ owing to its geometric size. In our study, the measurement was not restricted by the field size because the reference condition for measurement was 10 × 10 cm^2^.

From the gantry angle dependency, it was found that the deviation at 180° compared with that at 0° aligned well within − 0.86% of the stealth detector, while the ion chamber was − 2.74%. The conclusion derived from this observation is that the uncertainty associated with the ion chamber's setup influences accuracy. In particular, the procedure for measuring the output constancy with respect to the gantry angle has a significant effect. When using the existing setup, it is difficult to distinguish between the real deviation according to the gantry angle from an error. This is due to the positional uncertainty of the ion chamber setup with the laser attached to the wall of the treatment room to indicate the isocenter. It should be recognized that using only a gantry-mounted-type detector can prevent spurious errors from setup uncertainty.

The results of statistical analysis reveal a lack of statistically significant disparities between the two chambers concerning the linearity (output constancy) of X-ray monitor units and electronic monitor units. However, conspicuous distinctions emerged between the chambers in the context of dose variations versus X-ray output constancy and gantry angle versus X-ray output constancy. These findings contribute to an evaluation of the comparative performance of the Stealth chamber and FC65-G, imparting valuable insights for the enhancement of the QA process. Notably, the observed correlation between gantry angle and output suggests its potentially substantial influence on the precision of patient treatments.

The charge of the Stealth chamber is linearly affected by the field size. The charge for the field size investigated by the manufacturer and the value obtained by dividing the charge by the field size are shown in Table 10. If the annual QA is changed to a Stealth chamber, an output factor considering the field size must be obtained and used in the RTPS.

**Table 10:** The result of dividing the charge value and charge by the field size investigated by the manufacturer. High voltage bias used for measurement is − 400 V.

Field size (cm^2^)	Charge (nC)	Charge/field size (nC/cm^2^)	%diff
25 × 25	18,634	29.81*	
15 × 15	7132	31.70	6.3
10 × 10	3166	31.66	6.2
5 × 5	724	28.97	− 2.8
3 × 3	258	28.71	− 3.6
1 × 1	32	31.71	6.3

*Reference: Charge/field size = 29.81(nC/cm^2^).

When determining the output factor, it is important to consider the structural distinctions between the Stealth chamber and the CC-13 ionization chamber. Particularly in scenarios characterized by exceedingly high dose per pulse conditions, the phenomenon of ion recombination can exert a noteworthy influence on the chamber's responsiveness. Specifically, the CC-13 ionization chamber possesses a smaller activation area in comparison to the Stealth chamber. Consequently, the ion recombination correction coefficient may deviate from unity due to constraints related to charge collection efficiency. This occurrence can magnify the corrections applied to the measurements, thereby resulting in disparities between the observed output factor and the anticipated or expected ones.

The volume recombination dependence on per-pulse dose, particularly at low collection voltages, can be effectively approximated by theoretical models employing effective parameters. However, substantial disparities become evident at elevated collection voltages, as indicated in reference^26^. Studies involving FLASH-RT, employing high dose rates (HDRs), often utilize independent dose rate dosimeters, predominantly radiochromic films, as reported in literature^27^. Enhancement of ion collection efficiency and mitigation of polarization effects can be achieved by augmenting the electric field within the ionization chamber through the application of higher bias voltages or the reduction of electrode distances, as highlighted in previous research^28^.

Utilizing an ionization chamber designed to accurately assess high doses, such as FLASH-RT, is of critical significance, as highlighted in references^28, 29^. Studies have been conducted to compare measurements obtained with graphite calorimeters and charge measurements acquired from a plane-parallel ionization chamber to ascertain absolute collection efficiencies and deduce ion recombination factors^30^.

The Stealth chamber is located close to the linac head and operates in the HDR or ultra-high dose rate (UHDR) regime, breaking away from the traditional ionization chamber model. Following the approach of prior research, an assessment of the suitability of the Stealth chamber for high dose rate quality assurance will be pursued through a comparison with the existing chambers.

The present study examined the utility of Stealth chambers in the context of annual QA assessments. The raw data for the output results for each chamber, statistical comparison results, output factor, and reference figures for the setup conditions can be found in the supplementary materials (Supplementary data 1–5). Furthermore, future research endeavors aim to gauge output parameters for daily and monthly QA protocols, in addition to conducting QA evaluations for SRR/SBRT treatment modalities, with particular emphasis on scenarios involving high MU.

## Conclusion

This study was significant in that it demonstrated that transmission detectors have applications beyond their original purpose. In the present investigation, an assessment was made of the potential of Stealth chambers to streamline the annual QA process. The focus was on mitigating avoidable setup errors and consequently reducing the time and labor demands linked to QA procedures.

Except for the results for the QA item of output constancy with gantry angle, the results obtained using the Stealth chamber aligned (within 1.0%) with those obtained using the FC65-G. In addition, Stealth chamber was very easy to apply to small fields.

### Supplementary Information

Supplementary Information 1.

Supplementary Information 2.

Supplementary Information 3.

Supplementary Information 4.

Supplementary Information 5.

## Data Availability

The datasets generated during and/or analyzed during the current study are available from the corresponding author on reasonable request.
