# Supplementary material for: Dosimetric verification of annual quality assurance for a linear accelerator using a transmission type detector
Source: Sci Rep. 2023 Oct 21;13:17994. doi: 10.1038/s41598-023-45114-2 (PMC10590446; doi:10.1038/s41598-023-45114-2)
Supplement: Supplementary file 5 — Supplementary Information 5. [file 41598_2023_45114_MOESM5_ESM.pptx]

## Slide 1
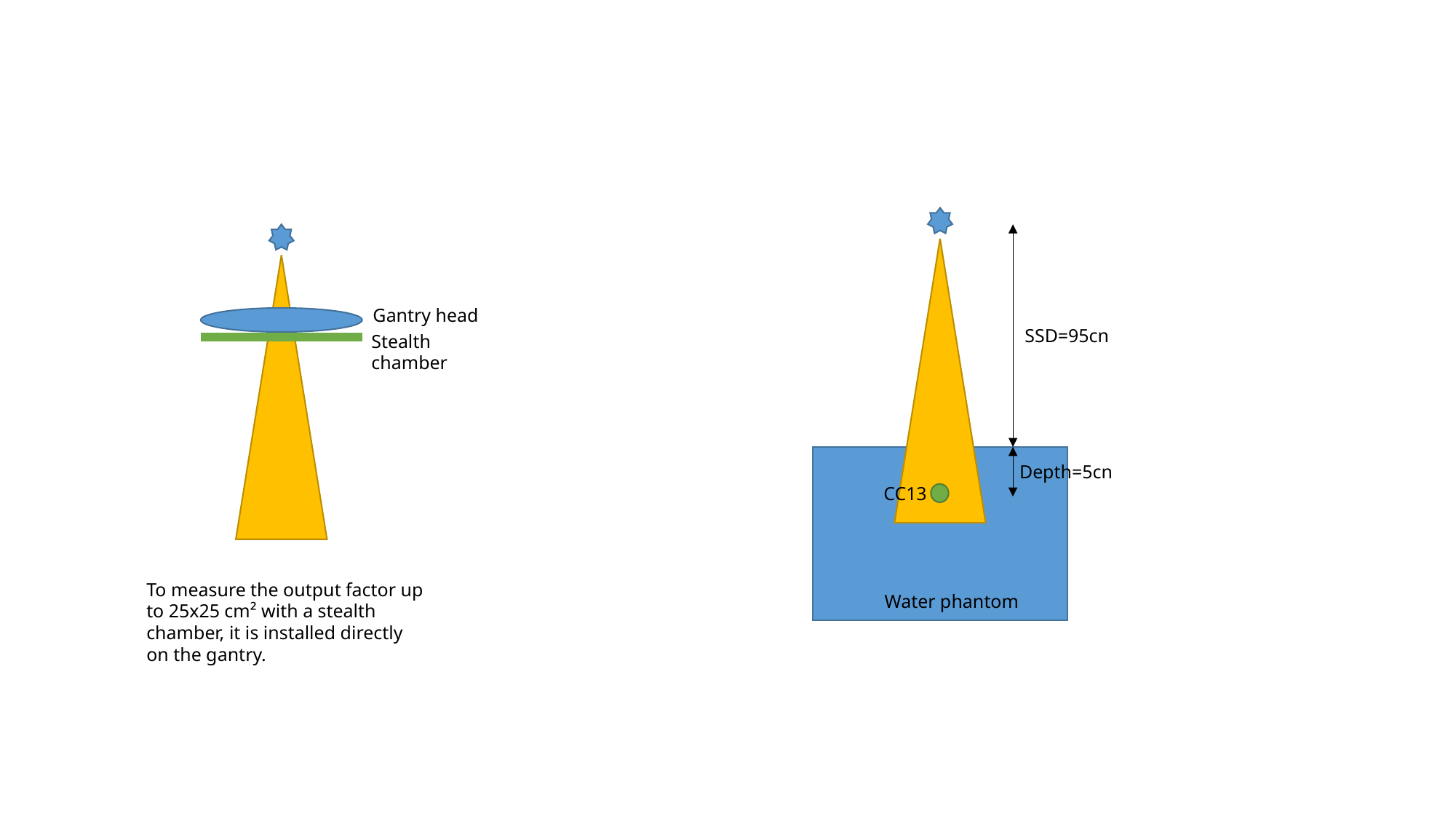

Gantry head
SSD=95cn
Stealth chamber
Depth=5cn
CC13
To measure the output factor up to 25x25 cm² with a stealth chamber, it is installed directly on the gantry.
Water phantom
